# The Interplay Between Affective Processing and Sense of Agency During Action Regulation: A Review

**DOI:** 10.3389/fpsyg.2021.716220

**Published:** 2021-09-16

**Authors:** Jakob Kaiser, Madalina Buciuman, Sandra Gigl, Antje Gentsch, Simone Schütz-Bosbach

**Affiliations:** LMU Munich, Department of Psychology, General and Experimental Psychology, Munich, Germany

**Keywords:** sense of agency, emotions, cognitive control, feedback processing, action regulation

## Abstract

Sense of agency is the feeling of being in control of one's actions and their perceivable effects. Most previous research identified cognitive or sensory determinants of agency experience. However, it has been proposed that sense of agency is also bound to the processing of affective information. For example, during goal-directed actions or instrumental learning we often rely on positive feedback (e.g., rewards) or negative feedback (e.g., error messages) to determine our level of control over the current task. Nevertheless, we still lack a scientific model which adequately explains the relation between affective processing and sense of agency. In this article, we review current empirical findings on how affective information modulates agency experience, and, conversely, how sense of agency changes the processing of affective action outcomes. Furthermore, we discuss in how far agency-related changes in affective processing might influence the ability to enact cognitive control and action regulation during goal-directed behavior. A preliminary model is presented for describing the interplay between sense of agency, affective processing, and action regulation. We propose that affective processing could play a role in mediating the influence between subjective sense of agency and the objective ability to regulate one's behavior. Thus, determining the interrelation between affective processing and sense of agency will help us to understand the potential mechanistic basis of agency experience, as well as its functional significance for goal-directed behavior.

## Introduction

To effectively reach our goals, it is important to assess how much influence we have over our environment. Sense of agency is the subjective feeling of being in control of one's actions and their perceivable effects (Gentsch and Schütz-Bosbach, 2015; Haggard, 2017). An inflated sense of agency has been associated with irrational and potentially self-destructive actions. For example, gambling addicts can have an unrealistically high feeling of control over chance outcomes (Orgaz et al., 2013). A diminished sense of agency has been related to inaction and a lack of perseverance (Bhanji et al., 2016; Studer et al., 2020). Strong feelings of loss of control have been associated with depression and anxiety disorders (Gallagher et al., 2014; Maier and Seligman, 2016). Thus, it is important to determine how sense of agency is established and through which mechanisms it influences our behavior.

A fundamental goal of instrumental actions is to receive positive outcomes and to avoid negative consequences. Therefore, we appear to constantly monitor the affective value of action outcomes. Since affective feedback is crucial for self-determined actions, it has been proposed that our sense of agency is bound to the processing of affective information (Synofzik et al., 2013; Gentsch and Synofzik, 2014; Ly et al., 2019). However, most previous research so far focussed on non-affective, sensory, and cognitive determinants of sense of agency. As a consequence, the potential functional relevance of affective processing for agency experience is not yet clearly understood.

This article aims to give an overview of current research concerning the interplay between affective processing and sense of agency. More specifically, we will discuss the empirical evidence regarding two questions (1) Do affective information or emotional states exert an influence on sense of agency? (2) Does sense of agency influence the processing of affective information and, particularly, on how humans process affective feedback during goal-directed actions? To preview our conclusions, current findings provide evidence for a bidirectional relation between affective processing and sense of agency. However, many details of the potential interaction between affect and agency experience still need to be clarified.

In the last part of this article, we will discuss the potential practical implications of the link between sense of agency and affective processing. This discussion will be guided by a tentative model about the interrelation between sense of agency, affective processing, and action regulation. For the purpose of this review, we define action regulation as the goal-oriented adjustment of ongoing or habitual behavior in response to environmental demands. In a nutshell, we propose that affective processing could play a role in mediating the influence between subjective sense of agency and the objective ability to regulate one's behavior. While an enhanced sense of agency might facilitate action regulation by heighten one's sensitivity toward task-relevant affective feedback, a diminished sense of agency in contrast could lead to blunted processing of affective feedback, resulting in less effective behavioral regulation. While more empirical findings are needed to critically evaluate this model, investigating the relationship between sense of agency and affective processing could help to elucidate the role of sense of agency for goal-directed behavior.

## Determinants of Sense of Agency

A number of different psychological terms are used in the literature to describe the subjective feeling of having or lacking agency over one's actions and the environment, such as sense of agency, self-efficacy, control beliefs, illusion of control, or learned helplessness (Ly et al., 2019). Control beliefs have sometimes been assessed as personality traits, meaning that people can maintain relative stable assumptions about their general degree of control over the environment (Craig et al., 1984; Galvin et al., 2018). In contrast, sense of agency is commonly meant to describe a psychological state, which can potentially fluctuate over time (Moore, 2016). For example, having success in learning a new skill can lead to a gradual increase in sense of agency (van der Wel et al., 2012). Accordingly, this article will focus on studies which manipulate or measure changes in agency experience in an experimental setting. For investigations of the relation between trait control beliefs and affect see for example: Gallagher et al. (2014), Harnett et al. (2015), and Koffer et al. (2019).

Previous research has identified many perceptual and cognitive factors which can increase subjective sense of agency. For example, participants are more likely to assume agency over sensory effects in the environment, if these effects follow their own actions in a predictable way and in close temporal proximity (Haggard and Tsakiris, 2009; Gentsch and Schütz-Bosbach, 2015). Thus, in many circumstances, sense of agency for our action is based on perceptual and cognitive processes.

The results of our actions often have personal relevance. Being able to produce perceivable sensory effects with our own actions can feel inherently pleasurable or motivating (Eitam et al., 2013; Karsh and Eitam, 2015). Moreover, we often engage in actions because we believe they might lead to rewarding or pleasant consequences, or they might help us to avoid punishment or detrimental outcomes. For example, during reinforcement learning, positive or negative feedback is usually provided after each action to either reinforce or discourage our current behavior (Cockburn et al., 2014; Kaiser et al., 2021). These types of action outcomes are not merely sensory events, but they can evoke affective states. One common approach to classify affective stimuli or states is to distinguish between positive and negative valence (Russell, 2003; Posner et al., 2005). In the context of goal-directed actions, action outcomes with positive valence occur when our actions lead to events which we perceive as pleasant or desirable, such as reward or praise. Action outcomes with negative valence are action effects which participants perceive as unpleasant or aversive, such as error messages or monetary losses. In the following review, we will summarize in how far the positive or negative valence influences sense of agency and, conversely, how sense of agency can influence the affective processing and affective states, such as the positive or negative feelings of participants.

## Does Affect Influence Sense of Agency?

This section will summarize experiments which tested the influence of affective context or emotional stimuli on sense of agency. More specifically, the guiding question is: Does positive compared to negative affect lead to an increase or decrease in sense of agency? Relevant studies manipulated affect-related aspects of an experimental task while measuring participants' sense of agency. Two types of affective manipulations were commonly used. Most studies manipulated the affective value of action effects, for example by letting participants perform an action which either led to the appearance of positive or negative action outcomes. This allowed to measure participants' sense of agency over positive compared to negative action effects. Some studies manipulated the affective context of an otherwise neutral action-effect sequence, for example via mood induction directly prior to an action. This allowed to test if participants' feelings can bias their sense of agency, even when affect is incidental to the action and its effect in question.

For measuring sense of agency, studies either employed explicit or implicit approaches. Explicit measures rely on self-report of agency experience, for example by asking people to rate their own perceived feeling of control over the outcome of each trial. It has been suggested that explicit self-report of agency might not always be accurate, e.g., because of demand effects or hindsight biases (Synofzik et al., 2008; Haggard, 2017). Therefore, some studies rely on implicit measures which avoid self-report. The most common implicit measures of sense of agency are sensory attenuation and temporal binding. Sensory attenuation describes the phenomenon that self-produced compared to passively perceived sensory effects lead to lower perceptual and neural impact (Blakemore et al., 2000; Gentsch et al., 2012). Temporal binding is a perceptual bias in which the delay between an action and the ensuing effect (e.g., a button press and a subsequent sound) is perceived to be shorter in time when the action is performed by oneself than by someone else (Haggard et al., 2002; Wolpe and Rowe, 2014). Based on these phenomena, many studies assume that stronger sensory attenuation or temporal binding indicate an increase in sense of agency. Since studies employing either explicit or implicit measures found partially divergent results, we will discuss relevant findings separately for explicit measures (i.e., self-report) and implicit measures (intentional binding or sensory attenuation).

### Influence of Affective Manipulations on Self-Report of Agency

Most studies using self-report measures found that positive compared to negative action outcomes lead to increased sense of agency. This has been shown for different types of affective stimuli such as consonant/dissonant sounds (Barlas and Obhi, 2014; Barlas et al., 2017), emotional facial expressions (Gentsch et al., 2015), and performance feedback in gambling tasks (Kulakova et al., 2017; Herman and Tsakiris, 2020) or motor control tasks (Oishi et al., 2018, 2019; Le Bars et al., 2020). The finding of increased sense of agency for positive action effects has been interpreted as part of a self-serving bias in human cognition (Gentsch and Synofzik, 2014; Haggard, 2017). Humans are more likely to attribute positive than negative events toward themselves (Mezulis et al., 2004). Thus, positive outcomes are more likely to be associated with increased sense of agency.

The studies described so far manipulated the affective valence of action effects by presenting either positive or negative action outcomes. Another approach to investigate the influence of affect on sense of agency would be to directly manipulate participants' current mood states. However, there is little evidence that mood manipulations influence explicit sense of agency. One study found that the induction of stress via the Trier Social Stress Test had no effect on agency ratings for otherwise neutral action effects (Stern et al., 2020). Since stress is usually experienced as a strongly negative affective state, this suggests that participants do not necessarily integrate their current feeling state in explicit agency judgements. More studies are needed to clarify if the affective context of an action might bias agency experience for unrelated, neutral action outcomes.

### Influence of Affective Manipulations on Implicit Agency Measures

Several studies tested if the valence of action outcomes has an effect on implicit measures of agency. For intentional binding, the evidence for affect-specific influences is mixed. Some studies found that negative compared to neutral or positive action outcomes decrease temporal binding (Takahata et al., 2012; Yoshie and Haggard, 2013; Barlas and Obhi, 2014; Borhani et al., 2017; Haggard, 2017; Nataraj et al., 2020). Since less temporal binding is assumed to indicate lower sense of agency, these findings are consistent with studies employing explicit agency measures, which found that negative action outcomes were associated with lower sense of agency.

However, there are also a number of studies which did not find any effect of outcome valence on temporal binding (Barlas et al., 2017, 2018; Kulakova et al., 2017; Moreton et al., 2017; Herman and Tsakiris, 2020). The absence of valence-specific binding effects in some studies might indicate that the valence of action outcomes only influences temporal binding under specific circumstances. In line with this assumption, a few experiments found that the effects of outcome valence on temporal binding depend on the predictability of action effects. Some studies reported that positive compared to negative outcomes only led to stronger temporal binding when the task context allowed to reliably predict if an action would lead to a positive or negative effect (Yoshie and Haggard, 2017). In contrast, when the valence of action outcomes was not predictable, no valence-specific binding effects were found. However, other studies found the opposite pattern of results, with increased binding for positive effects only for unpredictable, but not for predictable, outcomes (Christensen et al., 2016; Tanaka et al., 2020). Lastly, one study reported increased binding for predictable compared to unpredictable electric shocks (meaning strongly negative stimulation) as effects of one's own actions (Beck et al., 2017). Overall, these studies might indicate that the impact of outcome valence on agency interacts with other factors, such as anticipation and stimulus predictability. However, the exact nature of this interaction is not consistent across studies, and therefore not clearly understood.

Some experiments found that the valence of action effects might not only influence temporal binding between the action and the ensuing effect itself but could also have an impact on subsequent actions in the same task. For reinforcement learning tasks, it was found that negative compared to positive performance feedback on a trial increased intentional binding for actions on the subsequent trial (Di Costa et al., 2018; Majchrowicz et al., 2020). Errors are known to evoke increased top-down control of one's behavior to improve performance on subsequent trials (Ullsperger et al., 2014). Therefore, stronger binding after errors could indicate that engaging in top-down control is related to an enhanced sense of agency (Majchrowicz et al., 2020).

Only very few studies measured the effect of affective valence on sensory attenuation with mixed results. Some found stronger sensory attenuation for positive compared to negative action outcomes (Gentsch et al., 2015), others reported evidence for stronger attenuation for more negative action effects (Borhani et al., 2017; Osumi et al., 2019; Majchrowicz and Wierzchoń, 2021), or no effect of outcome valence on attenuation (Beck et al., 2017). Thus, it is currently not clear under which circumstances the valence of action outcomes modulates sensory attenuation.

As for explicit measures, there are less studies about the influence of participants' mood state on implicit measures of sense of agency for neutral action-effect sequences. Some studies reported that positive mood inductions prior to actions can increase temporal binding, while negative mood inductions led to decreased binding effects (Aarts et al., 2012; Obhi et al., 2013; Christensen et al., 2019). This could be seen as evidence suggesting that participants' affective state can bias their feeling of agency on an implicit level, with positive compared to negative mood increasing sense of agency.

### Summary: Influence of Affect on Sense of Agency

To summarize, several studies measured the effect of positive or negative action outcomes on sense of agency. Experiments relying on self-report show a mostly consistent pattern: Positive compared to negative action outcomes increase the explicit feeling of agency. For studies employing implicit measures the results are more varied and partly contradictory. There is evidence that positive compared to negative action outcomes either increase, decrease, or do not influence implicit sense of agency. At the very least, this indicates the need to identify additional factors which determine the impact of affective information on temporal binding and sensory attenuation. Importantly, there is evidence that sensory attenuation and temporal binding can be influenced by other factors than personal agency, such as the temporal predictability of action effects or changes in attention (Buehner and May, 2003; Kok et al., 2012; Kaiser and Schütz-Bosbach, 2018). Thus, it is not clear in how far the divergent results found via sensory attenuation or temporal binding capture genuine differences in agency experience, rather than confounding factors specific to the implicit measures itself.

There are very few reports about the influence of affective context, such as participants' mood, on sense of agency for neutral action effects. Some studies, mostly relying on temporal binding, suggest that positive compared to negative mood might increase sense of agency for unrelated action effects. It remains to be seen if similar effects can be found for explicit measures of agency. Moreover, future studies could consider the possibility that the impact of affective states on sense of agency depends on interindividual differences in affective processing. For example, individuals with diminished emotional coping skills might be more likely to infer agency from their current feelings.

## Does Sense of Agency Influence Affective Processing?

The following section will discuss experiments about the influence of agency experience on affective processing. Several approaches exist for the experimental manipulation of sense of agency (cf. Box 1). Most studies concerning the influence of agency experience on affect manipulated agency by varying the degree of choice (choice agency) or the degree of outcome reliability (outcome agency). For example, many studies compared the impact of rewards or losses which were either the result of forced-choice or free-choice actions. Such experiments allow measuring the effect of high compared to low sense of agency on affect-related measures. Two types of measures can be distinguished. First, some studies investigated the effect of agency manipulations on participants' affective state, for example by testing if changes in sense of agency influenced participants' mood. Second, other studies investigated the effect of agency manipulations on participants' sensitivity for stimuli with positive or negative valence. This allowed to test whether high compared to low sense of agency increased the subjective or neural impact of affective feedback. Answering this question would help to clarify if sense of agency influences the way we process affective information, such as positive or negative feedback during performance tasks. We will first discuss studies investigating agency effects on participants' affective states, and subsequently summarize experiments dealing with the influence of agency on the sensitivity for affective feedback.

Box 1 **Experimental manipulations of sense of agency.**

**Different techniques:** Experimental manipulations of agency typically aim at inducing a high or low sense of agency in participants to investigate the effect of agency experience on other psychological measures of interest. Techniques to manipulate agency can target different aspects of goal-directed behavior, and therefore differ widely across studies. At least three different types of manipulations can be distinguished:

- **Motor agency**: Many studies investigate agency at the level of motor executions, for example by comparing a condition where participants actively elicit a motor action to produce a sensory effect (high motor agency), with a condition where they just passively perceive the same effect (low motor agency; e.g., Baess et al., 2011; Kaiser and Schütz-Bosbach, 2018). Thus, agency in this case means to trigger an outcome with one's own motor action.
- **Choice agency**: Some studies manipulate the degree of choice over what type of action participants perform, for example by comparing a condition where participants can choose one of several buttons to press (free choice), with a condition where they have to press a predetermined button (forced choice; e.g., Fujiwara et al., 2013; Chambon et al., 2020). Agency in this case means to be able to choose between different actions with potentially different outcomes.
- **Outcome agency**: Since our actions are usually aimed at producing specific effects, such as obtaining rewards, we are more likely to feel in control when we can reliably produce the desired outcome (Moscarello and Hartley, 2017; Ly et al., 2019). Accordingly, some experiments manipulate agency experience by ensuring either that it is possible to produce a positive outcome (e.g., via highly reliable action-effect contingencies; high outcome agency), or giving participants no reliable chance to achieve the desired outcome (e.g., via random action-effect contingencies, low outcome agency; e.g., Nataraj et al., 2020; Li et al., 2021). Agency here means the ability to influence the environment in a way which is desirable to the agent.

**Real vs. illusionary agency:** Sense of agency is a subjective state, which can deviate from our objective level of control. Thus, sense of agency can be induced via real or illusionary agency. Inducing real agency means to provide an actual degree of control, for example by providing meaningful choices in a task. Inducing imaginary agency means to create an illusion of control, for example by making participants believe that outcomes in a task are dependent on their actions when in fact they are predetermined by the experimenter (e.g., Lorenz et al., 2015; Mühlberger et al., 2017). While providing an actual degree of control can lead to a more realistic task setting, inducing only the illusion of control might allow to more clearly attribute any experimental effect to changes in participants' subjective sense of agency, rather than other effects related to their objective mastery over the task.

**Do different agency manipulations target the same processes?** In many practical tasks, different aspects of agency are confounded. Importantly, it is unclear in how far different types of agency manipulations target the same or different cognitive and neural mechanisms. For example, a recent study reported that, compared to a condition where participants passively received rewarding outcomes (no agency), the neural processing of rewards was enhanced when participants performed a freely chosen action which triggered the rewarding outcome (motor and choice agency), but not when they had to perform a predetermined action to obtain the same reward (motor agency only; Hassall et al., 2019). This suggests that choice agency compared to motor agency might have different effects on the neural processing of action outcomes. More research is needed to clarify the potential differentiation between sense of agency on the level of motor execution (motor agency), action selection (choice agency), or outcome contingencies (outcome agency).

### Influence of Sense of Agency on Affective States

Several studies tested if sense of agency influences participants' self-reported emotional states. Most experiments found that having a degree of choice over one's actions and/or a feeling of control over ensuing action effects led to more positive or less negative affect (Abelson et al., 2008; Thuillard and Dan-Glauser, 2017, 2020; Stolz et al., 2020; Li et al., 2021). Moreover, participants prefer tasks which allow them to make choices compared to tasks where they cannot choose between different options, even when their own choices are not more likely to result in better outcomes (Leotti and Delgado, 2011, 2014; Fujiwara et al., 2013; Cockburn et al., 2014; Mistry and Liljeholm, 2016; Bobadilla-Suarez et al., 2017; Wang and Delgado, 2019). Items which are obtained through one's own choice are subjectively judged as being more valuable (Fujiwara et al., 2013). On a neural level, the mere anticipation of being able to make a choice has been found to increase activity in brain regions which are linked to reward processing, such as the ventral striatum (Tricomi et al., 2004; Bjork and Hommer, 2007; Leotti and Delgado, 2014; Lorenz et al., 2015; Romaniuk et al., 2019; Wang and Delgado, 2019; Stolz et al., 2020). Overall, these studies suggest that increased sense of agency is commonly experienced as desirable, and leads to increased positive affect (Leotti et al., 2010).

While having some degree of choice can increase positive affect, being presented with too many options can lead to increased negative, not positive, feelings (Iyengar and Lepper, 2000; Reutskaja and Hogarth, 2009). Having to consider a high number of different options might lead to information overload and, thus, higher cognitive demand (Scheibehenne et al., 2010; Chernev et al., 2012). Thus, the positive effects of choice agency can potentially be diminished or even be reversed in contexts where increased freedom of choice significantly increases task difficulty (Greifeneder et al., 2010).

Several studies investigated the influence of sense of agency on neural or subjective measures of pain. Most of these experiments provided participants with some (real or illusionary) possibility to control the presence or duration of painful stimulation. Compared to a condition where participants experienced the same degree of pain stimulation without any form of control, the feeling of having agency usually led to lower self-reported levels of pain intensity, as well as less activity in brain areas associated with pain processing (Salomons et al., 2004, 2014; Wiech et al., 2006; Vancleef and Peters, 2011; Mohr et al., 2012; Szczepanowski et al., 2013; Bräscher et al., 2016). While pain is usually not considered to be an affective state, it is commonly associated with strong negative affect. Therefore, these findings are consistent with the notion that increased sense of agency can lower negative affect.

To conclude, most studies indicate that heightened sense of agency increases positive and/or decreases negative affect. However, an overabundance of choice might lead to aversive affective reactions in contexts where the decision-making process strongly increases task demand.

### Influence of Sense of Agency on the Processing of Affective Stimuli

Several studies investigated if sense of agency increases or decreases the sensitivity for affective stimuli. Most experiments concerned with this question manipulated participants' sense of agency for positive or negative task feedback during learning or gambling tasks, while measuring neural correlates of feedback sensitivity via EEG. Commonly used measures entailed ERPs like the reward positivity component, a midcentral positive deflection which tends to be increased for positive compared to negative feedback (Proudfit, 2015). This component is also often reported as error negativity, which is calculated as the difference in reward positivity between negative and positive stimuli (Mühlberger et al., 2017). Other studies measured the P300 or oscillatory midfrontal theta power, both of which tend to show increased activity during task-relevant expectation violations and errors (Polich, 2007; Kaiser et al., 2019).

Most studies reported that high compared to low agency increased the neural responses for affective action outcomes. This has been found for the reward positivity/error negativity component (Yeung et al., 2005; Bellebaum et al., 2010; Li et al., 2011; Martin and Potts, 2011; Bismark et al., 2013; Legault and Inzlicht, 2013; Bellebaum and Colosio, 2014; Meng and Ma, 2015; Mühlberger et al., 2017; Mei et al., 2018; Yi et al., 2018; Hassall et al., 2019; Fang et al., 2020; Zheng et al., 2020), as well as for the P300 (Bellebaum et al., 2010; Mühlberger et al., 2017; Mei et al., 2018; Yi et al., 2018; Hassall et al., 2019; Fang et al., 2020), and midfrontal theta power (Zheng et al., 2020). Overall, these findings suggest that sense of agency increases the neural impact of affective feedback.

Studies reporting that sense of agency increases the neural impact for affective feedback might appear to be inconsistent with the phenomenon of sensory attenuation. As discussed above, sensory attenuation refers to the finding that sense of agency leads to lower, not higher, neural impact for self-produced action effects (Baess et al., 2011; Gentsch and Schütz-Bosbach, 2015). Importantly, sensory attenuation has most often been reported for non-affective action effects with little or no direct relevance for participants. In contrast, affective stimuli often have practical significance for humans. For example, positive or negative action outcomes can provide feedback over our current performance during goal-directed tasks. Thus, sense of agency might increase the impact of affective and task-relevant, but not of non-affective incidental action effects, to highlight the most self-relevant results of our own actions.

Moreover, studies investigating agency effect for non-affective vs. affective stimuli tend to differ with respect to the type of agency manipulation (cf. Box 1): Sensory attenuation for non-affective stimuli has been mostly found when manipulating motor agency, usually by comparing passive perception with active production of sensory effects (Blakemore et al., 1998; Weiss et al., 2011). In contrast, neural enhancement for affective stimuli has most often been reported for studies which manipulated choice and/or outcome agency, for example by comparing free-choice with forced-choice tasks (Li et al., 2011; Mühlberger et al., 2017; Mei et al., 2018). Accordingly, the occurrence of neural attenuation compared to neural enhancement might partly be related to which type of agency (i.e., motor/choice/outcome) is being manipulated (Hassall et al., 2019).

Lastly, sensory attenuation was commonly assessed via early markers of sensory processing, such as the N100 component in EEG (Baess et al., 2011; Timm et al., 2016). In contrast, neural enhancement for affective stimuli was usually found for frontocentral indicators of reward and punishment processing, such as the midfrontal reward positivity or P300. Thus, we cannot exclude the possibility that sense of agency is more likely to lead to an attenuation of early neural markers of sensory impact, but an enhancement of neural activity related to evaluative processing.

Overall, most current studies show that sense of agency can increase the neural impact of affective stimuli. We still lack sufficient empirical data to fully explain the divergent findings between agency effects for non-affective vs. affective action effects. It will be important to determine under which circumstances increased sense of agency leads to neural attenuation compared to neural enhancement, for example by investigating the role of task-relevance (task-relevant vs. incidental action effects), type of agency experience (via independent manipulations of motor/choice/outcome agency), and the neural processing stage (by comparing effect on neural components related to early sensory vs. evaluative processing).

### Does Sense of Agency Lead to a Valence-Specific Bias in Neural Processing?

While many studies show that higher sense of agency increases the neural impact of affective feedback, it is less clear if these agency-related effects on affective processing are equally strong for positive and negative stimuli. Determining if agency experience leads to a selective enhancement of either positive or negative feedback is important, because such a finding would imply that sense of agency generates a valence-specific processing bias. One study found behavioral evidence for an agency-related positivity bias in a reinforcement learning task. High compared to low sense of agency led to selective increases in learning rates after positive, but not negative, feedback (Chambon et al., 2020). Such a selective enhancement of positive feedback could help to explain self-serving biases in the evaluation of one's own actions (Mezulis et al., 2004).

On a neural level, the evidence that sense of agency induces a valence-specific bias is less conclusive. Many studies do not test for potential valence-specific effects of sense of agency. Some of the experiments which address this question find that agency affects the neural processing of both positive and negative feedback to a similar degree (Mei et al., 2018; Hassall et al., 2019). However, others report that increased sense of agency more strongly enhances the neural impact of negative feedback (Bellebaum et al., 2010; Martin and Potts, 2011; Legault and Inzlicht, 2013), or positive feedback (Mühlberger et al., 2017). The inconsistency between studies might in part reflect the fact that studies concentrate on neural measures which are either more strongly related to reward sensitivity (midfrontal reward positivity) or the processing of errors and expectation violations (P300/midfrontal theta power). In line with this assumption, one study found that increased sense of agency led to an increased reward positivity component in the EEG for positive (but not negative) feedback, but increased midfrontal theta power for negative (but not positive) feedback (Zheng et al., 2020). This suggests that sense of agency increases the neural impact of both positive and negative feedback, albeit for different neural processes.

### Summary: Influence of Sense of Agency on Affect

To summarize, research indicates that heightened sense of agency increases positive affect. However, while free choice over some task-relevant aspects can be employed to induce increased sense of agency, an overabundance of choice options might intensify task complexity and thus lead to negative affect. Furthermore, while heightened sense of agency is commonly assumed to lead to lower neural impact for non-affective stimuli, it has been found to lead to increased neural impact of affective feedback. Further research is needed to determine if agency-related changes in neural processing of affective feedback occur for both positive and negative feedback to the same degree, or if agency induces a valence-specific bias in neural processing, in the sense of a selective increase in sensitivity for either positive or negative feedback.

## The Role of Sense of Agency And Affective Processing for Action Regulation

**Figure 1:**
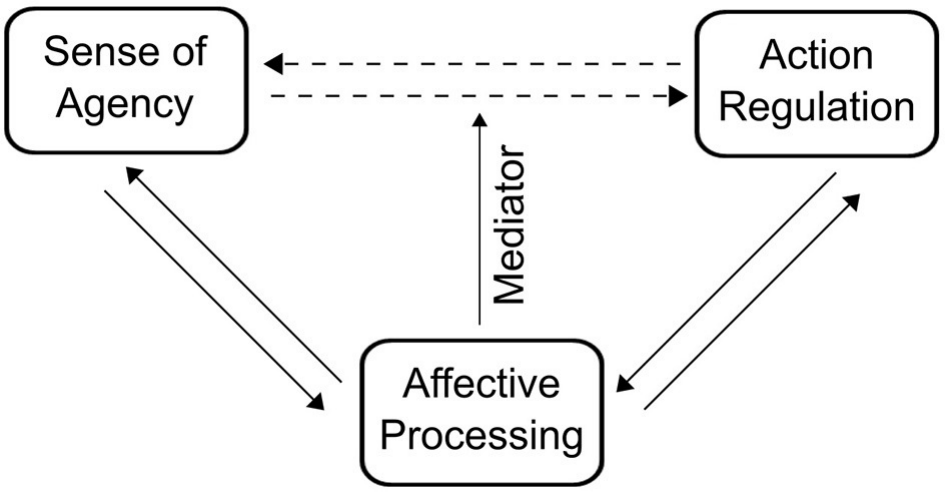
Schematic representation of the proposed relation between sense of agency, affective processing, and action regulation. The model assumes a bidirectional relation between affective processing and sense of agency, as well as between affective processing and action regulation. Importantly, affective processing partially mediates the influence between sense of agency and action regulation.

The previous sections have shown that current research provides evidence for a bidirectional relationship between sense of agency and affect-related processes. Changes in affective states are associated with changes in sense of agency, and changes in sense of agency can alter affective states and the processing of affective stimuli, such as positive or negative performance feedback. This section will discuss the potential functional implications of the interaction between affective processing and sense of agency. This discussion focusses on a tentative model of the relationship between sense of agency, affect, and action regulation (Figure 1).

Action regulation in this context refers to an adjustment of ongoing behavior in order to improve one's chances to successfully reach a goal. Successful action regulation commonly depends on voluntary exertion of cognitive control mechanisms to override goal-incompatible behavioral tendencies (van de Vijver et al., 2011; Gratton et al., 2017; Kaiser and Schütz-Bosbach, 2019, 2021). Concerning the role of affective processing, we specifically focus here on the processing of positive and negative performance feedback during tasks which necessitate action regulation. We suggest that the interaction between sense of agency and affective processing plays a role in this process, since changes in sense of agency can either increase or dampen the sensitivity for affective task feedback (Bhanji and Delgado, 2014; Mühlberger et al., 2017; Hassall et al., 2019). Since behavioral adaption relies on the accurate processing of affective feedback, agency-related changes in affective processing can facilitate or hinder feedback-guided action regulation. We will discuss the main aspects of this potential mechanism in this section.

### The Influence of Sense of Agency on Action Regulation

As illustrated in Figure 1, we assume a bidirectional relationship between subjective sense of agency and the objective ability to regulate one's actions and the environment. Under normal circumstances, being successful in regulating one's behavior according to one's current goals increases sense of agency (Moscarello and Hartley, 2017). Conversely, there is also evidence that subjective sense of agency can influence objective action regulation performance. Learned helplessness describes the phenomenon that the experience of having no control can lead to diminished performance in learning tasks (Maier and Seligman, 2016). Thus, low sense of control can have a detrimental effect on action regulation capacities.

Enhanced sense of agency has been related to better performance in tasks which require action regulation. For example, during motor learning tasks participants usually have to perform training sessions to learn challenging motor actions which require efficient or precise motor movements. Sense of agency during training can be induced by, for example, letting people choose the order of training tasks they have to perform. High vs. low sense of control has been found to lead to increased training success, meaning stronger improvements in motor performance (Sanli et al., 2013; Lewthwaite et al., 2015; Halperin et al., 2017; Iwatsuki et al., 2019; Iwatsuki and Otten, 2020; Matsumiya, 2021). Additionally, increased agency has been found to lead to lower error rates during cognitive control tasks (Legault and Inzlicht, 2013), and improved learning rates during memory tasks (Murayama et al., 2015; Murty et al., 2015). These findings suggest that sense of agency can facilitate action regulation.

### The Influence of Affective Processing on Action Regulation

Action regulation is often related to the processing of affective information: we tend to alter our behavior when it leads to negative results and repeat the same actions when they are followed by positive outcomes. Thus, the monitoring of positive or negative performance feedback is vital for behavioral adjustments (Ullsperger et al., 2014). Negative feedback can lead to cognitive and neural changes, such as increased activity in brain circuits involved in cognitive control, which facilitate changes of ongoing behavior (van Driel et al., 2012; Beatty et al., 2020; Kaiser et al., 2021). For example, the affective-signaling theory proposes that affect is an important component of the neural conflict monitoring system, with negative affect eliciting an increase in executive control resources (Dignath et al., 2020). On the other hand, positive feedback is known to elicit increased activity in reward-related brain areas, which can lead to a reinforcement of goal-compatible behavior (Holroyd and Coles, 2002; Krigolson, 2018). Therefore, sensitivity to affective feedback is assumed to be one determinant of action regulation success (van de Vijver et al., 2011; Kaiser et al., 2021). Diminished sensitivity toward affective feedback has been associated with maladaptive behavior, such as diminished self-control in daily life (Overmeyer et al., 2021). Additionally, blunted neural reactivity toward errors or reward feedback has been observed in several psychological disorders which are characterized by self-regulatory problems, such as substance abuse or pathological gambling (Euser et al., 2013; Gorka et al., 2019; Li et al., 2020). Accordingly, any psychological factor that significantly modulates the impact of positive or negative feedback could potentially influence action regulation performance.

### Affective Processing as a Mediator Between Sense of Agency and Action Regulation

As discussed above, several studies have found that high compared to low sense of agency is associated with changes in the processing of affective information, such as changes in the neural impact of affective feedback. Affective feedback is an important determinant of action regulation. Based on these findings, the model outlined in Figure 1 assumes that affective processing mediates the influence of sense of agency on action regulation. The proposed relationship in Figure 1 should be understood as a partial mediation, meaning that there are most likely other, non-affective mediators between agency experience and action regulation. For example, cognitive beliefs about one's self-efficacy might also partly predetermine regulative success (Sanli et al., 2013). Overall, we assume that increases in sense of agency lead to heightened neural sensitivity for affective feedback. Heightened sensitivity for affective feedback improves feedback learning and thus increases the chances to successfully self-regulate behavior. Conversely, low sense of agency could blunt sensitivity for affective feedback. This agency-induced decrease in feedback sensitivity could diminish feedback learning performance, thus having a detrimental influence on action regulation. Accordingly, affective processing could represent a specific mechanism which links subjective experience of agency with the objective ability to regulate one's behavior.

As discussed above, previous research provides ample evidence for a link between sense of agency and affective processing on the one hand (e.g., Leotti et al., 2010; Chambon et al., 2020; Zheng et al., 2020), and affective processing and action regulation on the other hand (e.g., Holroyd and Coles, 2002; Dignath et al., 2020; Kaiser et al., 2021). However, it should be noted that so far there are almost no empirical tests of the potential mediating role of affective feedback processing between sense of agency and action regulation. To the best of our knowledge only one study so far tested a closely related hypothesis: Legault and Inzlicht (2013) investigated the effect of feeling of autonomy on the performance in a cognitive control task. Autonomy was induced by providing an illusion of choice over the task, meaning that their operationalization of autonomy effectively manipulated choice agency. It was found that illusion of choice led to lower error rates, indicating improved action control. Importantly, the increase in performance for participants with choice agency was statistically mediated by stronger neural reactions toward error feedback, as measured via the feedback-related negativity component with EEG. It was concluded that increased error sensitivity mediates the relation between the feeling of autonomy and action control. This finding is consistent with the proposed interrelation between sense of agency, affective processing and action regulation.

Interestingly, Legault and Inzlicht (2013) found a mediation effect selectively for neural reactivity toward negative, but not positive feedback. This suggests that sense of agency influences action regulation by selective increases in error sensitivity. However, as discussed above, there are inconsistent results regarding sense of agency selectively boosting the processing of positive feedback (Mühlberger et al., 2017; Chambon et al., 2020), negative feedback (Bellebaum et al., 2010; Legault and Inzlicht, 2013), or both (Zheng et al., 2020). Accordingly, it remains an open question if the mediation of agency effects on regulation performance is primarily driven by changes in positive or negative feedback processing. Overall, due to the lack of more empirical reports regarding this question, the proposed mediating role of affective processing between sense of agency and action regulation remains tentative. However, we believe that investigating this link will be a promising avenue to develop a mechanistic understanding of the interaction between agency experience and goal-directed behavior.

## Conclusions and Future Directions

To summarize, experimental research indicates a bidirectional relation between sense of agency and affective processes. Several studies found evidence that emotional stimuli and/or affective states can, to a certain extent, have an influence on sense of agency. Conversely, manipulations of sense of agency have been shown to be associated with changes in affective states, as well as changes in the processing of affective information. Since the processing of affective information, particularly positive and negative performance feedback, is crucial for learning and action regulation, affective processing represents a potential link between the subjective feeling of being in control and the actual ability to gain control over one's actions and the environment. Our review has identified several questions which need to be clarified to fully understand and specify the bidirectional interrelation between sense of agency and affective processing, as well as its functional implications for action regulation.

For determining the influence of affective information on sense of agency, it would be important to clarify the discrepancy between affect-related effects on implicit measures of agency. While most studies employing self-report measures find that positive compared to negative affect increases sense of agency, experiment using implicit measures such as sensory attenuation or intentional binding come to diverging conclusions about the influence of affect on sense of agency. This suggests that emotional effects on implicit measures depend on additional variables which have not yet been clearly identified (but see Beck et al., 2017; Yoshie and Haggard, 2017). Importantly, it needs to be determined in how far affect-related effects on measures such as temporal binding and sensory attenuation indicate genuine alterations in sense of agency, rather than the susceptibility of implicit measures to perceptual or cognitive influences which do not directly reflect agency experience (Buehner, 2012; Kaiser and Schütz-Bosbach, 2018).

Concerning the influence of sense of agency on affective processing, future studies need to distinguish valence-independent from valence-specific effects. Numerous studies show that high compared to low sense of agency increase the neural impact of affective feedback. It is less clear in how far agency-induced changes in affective processing reflect a general increase in sensitivity for performance feedback as compared to a processing bias for either positive or negative feedback. If sense of agency selectively increased neural sensitivity for positive feedback, this could help to explain the neural underpinnings of the self-serving attributional bias, meaning increased sensitivity for positive results of self-determined actions (Mezulis et al., 2004; Chambon et al., 2020). Conversely, if sense of agency increased sensitivity for negative feedback, this could potentially represent an adaptive mechanism to adjust one's behavior after self-produced errors.

Lastly, more research is needed on the functional implications of the link between sense of agency and affective processing. Sense of agency can sometimes boost or diminish performance during goal-directed behavior. Since agency experience modulates the impact of affective feedback, and affective feedback is crucial for behavioral adjustments, affective processing is a promising candidate for a mediating factor between sense of agency and action regulation (Legault and Inzlicht, 2013). However, this possibility needs to be investigated empirically.

It will be important to clarify the neural mechanisms that link affective processing and sense of agency. Potential candidate mechanisms include limbic structures in the basal ganglia, such as the ventral striatum which is involved in the processing of reward, and areas of the medial prefrontal cortex, which are assumed to play a role in the processing self-relevant information (Cockburn et al., 2014; Wang and Delgado, 2019). Some studies indicate that intercommunication between these areas might be related to changes in sense of agency due to affective performance feedback (Wang and Delgado, 2019; Stolz et al., 2020). Moreover, it is noteworthy that both emotional processing and sense of agency have been related to the processing of bodily information. Affective stimulation is often accompanied by peripheral physiological changes (Kreibig, 2010). At the same time, sense of agency is assumed to be related to the sense of body ownership, meaning the feeling of having and controlling one's own body (Asai, 2015; Braun et al., 2018; Gonzalez-Franco et al., 2020). Since both agency experience and affective experience might partially rely on bodily information, the role of bodily changes in linking these two processes could be an important point of consideration in future studies.

The potential relationship between affective processing and sense of agency could have implications for psychopathological conditions that are marked by distortions in agency experience, such as schizophrenia or depersonalization disorder (van Haren et al., 2019; Kozáková et al., 2020). For example, schizophrenia has sometimes been linked to a distorted processing of affective information (Rahm et al., 2015; Maher et al., 2016). For such disorders, it would be important to know if alterations in agency experience and affective processing might be related. Lastly, previous research has separately investigated the developmental trajectory of affective processing (Quinn et al., 2011; Hoemann et al., 2019) and the evolving sense of agency (Zaadnoordijk et al., 2020; Meyer and Hunnius, 2021). With respect to their potential interactions, future research could probe the question in how far developmental changes in the sense of agency and affective processing co-occur or are even functionally related.

To conclude, while most previous research focusses on non-affective sensory and cognitive determinants of sense of agency, there are numerous findings which indicate that affective processes play an important role in our agency experience. Future research needs to specify the interactions between affect and sense of agency, for example with regards to valence-specificity of agency-related effects, as well as the role of other contextual factors which determine the influence of emotional information on agency experience. Importantly, studying the relation between sense of agency and affective processing could be a crucial step in linking the subjective experience of agency to failure or success during goal-directed behavior.

## Author Contributions

JK, MB, and SG reviewed the literature. JK drafted the manuscript. AG and SS-B contributed to the manuscript. All authors contributed to the article and approved the submitted version.

## Funding

This work was supported by a grant to AG and SS-B by the German Research Foundation (DFG)—Project No. 402781060.

## Conflict of Interest

The authors declare that the research was conducted in the absence of any commercial or financial relationships that could be construed as a potential conflict of interest.

## Publisher's Note

All claims expressed in this article are solely those of the authors and do not necessarily represent those of their affiliated organizations, or those of the publisher, the editors and the reviewers. Any product that may be evaluated in this article, or claim that may be made by its manufacturer, is not guaranteed or endorsed by the publisher.
